# Dr. W. P. Dewes' Essay on Puerperal Convulsions

**Published:** 1820-06-01

**Authors:** 


### VIII.

*An Essay on Puerperal Convulsions.* By W. P. Dewes, of Philadelphia.

American Medical Recorder, No. III.

Socrates, whose mother was a midwife, was accustomed to observe, that he followed the same trade, excepting that it was with the opposite sex, whose brains he delivered at the proper period of encephalo-gestation. The Press may now be considered as the Accoucheur General of Authors. When the term of cerebral pregnancy is expired, and sometimes before this period, the intellectual bantling is ushered into the world by the groaning press, consigned to its Nurse, the Publisher—a Personage of no mean note in the Republic of Letters. Formerly, indeed, this nurse was believed to be the principal arbiter of the youngster's fate; and afterwards, the process of Christening, or *reviewing*, was looked upon as a terrible ordeal; but the world is now grown wiser, and the bookseller or critic can exert but a very limited and temporary influence on the fate of intellectual productions. The case indeed, is somewhat modified in *Medical* Literature. So small comparative ly is the number of those who have leisure or inclination to peruse medical works in the original, that, with a few exceptions, their circulation, *in propria forma*, is a sphere of extremely small diameter. The only objects then, which a medical writer need, or ought to have, in coming before the public, are — philanthropy towards his fellow creatures, and honest reputation for himself. To diffuse the knowledge of a book, there is not any engine more powerful than a periodical publication, conducted chiefly on analytical principles. In a very few months from the date of its stated periods, it traverses every parallel, from the Isthmus of Suez to that of Darien—from Van Diemen's land to Ultima Thulé, disseminating those sentiments of an author through all quarters of the Globe, which, but an instant before, were confined to a solitary individual.

The influence, then, of well conducted Journals, on the progress of medical science, is exceedingly great. The public are aware of this, as is clearly evinced by the circulation of the worst Journals compared with the best books. The English Medical Journals have a wider range than those of any other country, in consequence of the vast extent of our colonies, and the diffusion of our language over the great American territory. This brings us to the subject of the present paper. — The American Medical Recorder, in which it is published, is a Quarterly Journal, like our own, but differently arranged. We have put ourselves in direct communication with its Editor, for a mutual exchange; and we have reason to believe, that many of our Analytical Reviews will be republished in this very respectable transatlantic cotemporary;* and thus take a more general course of circulation in the Columbian Republic.

Dr. Dewes justly observes, that no disease is more formidable or menacing than puerperal convulsions; the attack of which is always ferocious, and too often fatal.

"The young practitioner, instead of becoming enlightened by consulting authorities, is bewildered by opposite opinions: he either pursues with reprehensible temerity the directions of one, or blameably temporises agreeably to the views of a second; or fatally urges the remedies of a third." 308.

Discordant views in therapeutics generally have their source in erroneous notions of pathology. Thus Lamotte, Puzos, Osborn, &c. believed that puerperal convulsions arose from irritation of the uterine fibres—others, as Hull, Garthshore, &c. conceived them to be epileptic—while Bland and some others, imagined them to be nervous.

"The first set make safety exclusively to consist in immediate delivery: the second deprecate the practice, and rely for success on the powers of Nature; while the third recommend the use, the fatal use of opium." 310.

Our author thinks that neither of the plans of treatment alluded to can be uniformly pursued without fatal consequences; and therefore he endeavours to remove ambiguity by a consideration of the causes, phenomena, distinctions, and cure of the different species of puerperal convulsions.

I. *Etiology.* This our author asserts to be immersed in complete darkness; he therefore only states a few of the opinions entertained by distinguished obstetric practitioners. Denman attributed the remote cause to a particular influence of the air, or some change made in the constitution by the customs and manners of civic and civilized life. Dr. Dewes' experience does not confirm this. During a country and city practice of twenty-five years, he found almost all his cases of puerperal convulsions among the lower class of people, and particularly among the robust and plethoric, rather than a-

* Dr. Burder's Essay on Syphiloid Diseases, the first article in our Quarterly Series, has been republished in the above work, and is now circulating widely through the United States.

mong the delicate and debilitated in the higher walks of life. When the disease occurred in the latter class, our author generally found the convulsions of the hysterical kind, which, as will be seen farther on, are not near so dangerous as the other species.

The pressure of the gravid uterus upon descending blood-vessels, causing a greater distribution of blood to the head, can hardly be regarded in the etiology of this disease, since this pressure is pretty uniform in every pregnancy; whereas convulsions are of comparatively rare occurrence.

Baudelocque considers "an extreme sensibility of the uterine fibres, a violent distention of the edge of the orifice of the uterus, and of the parts which form the entrance of the pudendum," as causes of convulsions. Dr. D. thinks that may sometimes be the case, but he doubts if it ever produces the kind that would, if left unattended to, have a fatal termination.

II. *Symptomatology*. In almost every case of convulsions during pregnancy, our author has observed the attack to be preceded by the following train of symptoms, differing more in intensity and duration, than in peculiarity.

"Head-ache, ringing in the ears, vertigo, and often a temporary loss of vision—these symptoms continue a longer or shorter time in different patients; some complaining of them many days, others but a few hours, and some only a few minutes before the convulsive paroxysm takes place.

"These symptoms so uniformly prevail before the convulsions come on, that I have almost constantly, where I have been consulted for severe head-ache, in women near their term, directed a liberal bleeding, with pretty smart purging; and by this anticipation, I have no question but that this formidable disease was prevented. There is a symptom which I have observed in several instances, that has never failed to be followed by convulsions, and they of the epileptic or apoplectic kind: this is a severe and intense pain in the middle of the forehead, resembling, as they expressed it, 'a nail driven into the head.' So certain have I always been that this was the prelude to convulsions, that I have uniformly and promptly used the lancet wherever I have been permitted; and, I firmly believe, with the most salutary effect." 314.

Some are said to complain of violent pain in the stomach, premonitory of the convulsive attack; and this is considered by Dr. Denman a more fatal symptom than head-ache. Our author observed, that when the head-ache or other premonitory symptom preceded, for a considerable time, the attack of convulsions, the disease was then milder, or more manageable, than in opposite circumstances. Dr. Dewes thinks that pregnant women may be seized with convulsions from other causes than gestation; and these convulsions are far more dangerous than when they proceed from pregnancy, as a remote cause.

"When pregnancy is instrumental in the production of this disease, it is almost always at that period, when the uterine fibres are at their greatest stretch, and when the os tincæ is about to dilate for labour, or where they suffer some peculiar irritation from the contents of the uterus, which has the same effect." 315.

Our author thinks that this disease is never preceded by an aura, as in epilepsy. After the patient has suffered a longer or shorter time from the above-mentioned symptoms, she is suddenly seized with quickly repeated spasms; the face and eyes are twitched with incredible quickness, in almost every possible direction; the arms, legs, and whole body, are violently agitated, one side being sometimes more affected than the other; the face becomes flushed, then livid; nay, black —the tongue is strongly thrust forward between the teeth, and is frequently severely wounded. The respiration is, at first, much hurried, but eventually becomes almost suspended; the carotids throb violently; the jugular veins are distended; "a peculiar noise is made by the mouth, not unlike what is termed 'a cat spitting;'* a froth issues from the mouth, generally tinged with blood; the pulse, in the beginning, is full, frequent and tense; but quickly becomes rapid, small, and eventually almost imperceptible; the urine and fæces are sometimes discharged; a cold clammy sweat bedews the whole body, and the fit then begins to subside. This, however, is gradual, and seldom suddenly at once; in short, the morbid symptoms recede, somewhat in the order in which they advanced; the patient, however, remaining, for the most part, insensible, or comatose—being with difficulty roused, and when she does recover her scattered senses, she is generally without recollection of what has passed. This truce is usually of short duration; convulsion succeeds convulsion, without our being able to determine the cause or period of their return.

When this disease attacks a woman actually in labour, or about to be so, we may observe a pretty strict recurrence of the fits with the probable return of the pains. Mean time, the face becomes very much swollen, particularly the eyelids and lips; indeed the whole body seems to partake of this intumescence, but not so conspicuously as the face. This

---

* There is something so characteristic in this noise, that Dr. Denman has declared he could tell the condition of the patient, though in another room.

swelling does not immediately subside with the convulsions which caused it; but remains generally for many days after they have disappeared. Dimness of sight, nay blindness for weeks, are no infrequent consequences of this disease.

Our author divides puerperal convulsions into three species, to which he gives the names (without meaning to defend the propriety of the *names*) of epileptic, apoplectic, and hysterical.

In the *first* species he believes the premonitory symptoms always shew themselves some days before the attack, and it is uniformly attended with a strong determination to the head, producing an engorgement of its vessels. It may come on at any period of pregnancy; but most frequently after the sixth month. This species almost always produces labour, or at least is accompanied by it. It may terminate favourably, when properly treated, or it may be converted into the apoplectic form.

In the *second* species we have nearly all the premonitory phenomena, but they are of much shorter duration. It may, like the former, occur at any period of utero-gestation, but does not necessarily produce or become attended by labour. Hence it would seem as if it were produced by causes somewhat independent of pregnancy; or, at least, that this last state is only the exciting cause of the disease.

In the *third* species we have not the same train of premonitory symptoms. If headache attend, it is not so severe or permanent. There is frequently a ringing in the ears, generally globus hystericus and palpitations of the heart. The face is much less convulsed, while the larger muscles of the body are much more powerfully agitated than in the other species. The patient is sometimes very obstreperous, and the muscles on the posterior part of the body are almost always so violently contracted, that our author has seen women raised up like an arch as in opisthotonos. There is no frothing at the mouth, or that peculiar sibilating noise which characterises the first species. After the fit, the patient can, for the most part, be roused to attention by a repetition of efforts for this purpose, or will frequently become coherent, as soon as she recovers from the fatigue or exhaustion occasioned by her violent struggles.

"After the fit has subsided a short time she will often open her eyes, and vacantly look about, and then, as if suddenly seized by some sense of shame, will sink lower into the bed, and attempt to hide her head beneath the bed clothes."

The pulse is also less disturbed in this species, which attacks, in preference, women of delicate habits, and who are habitually subject to hysteria.

*Treatment.* In the first species our great dependence must be placed upon bleeding, which must be done promptly and copiously, or no good can be expected. The blood, our author observes, should be abstracted as rapidly as possible, from a large vein, and by a large orifice, that the heart and vascular system may be quickly enfeebled. He is convinced that, in these cases, if blood be slowly drawn off, the arterial system, in consequence of being relieved of a part of its load, acts with renewed vigour, and augments the existing mischief. He justly observes that, *cæteris paribus,* the nearer the part affected we can bleed, the greater will be the relief afforded; hence, as dissection proves to us that the brain invariably suffers in this disease, to a greater or less extent, "we should lay it down as a rule, to bleed from the jugular vein or veins whenever practicable, or from the arm or arms, from large orifices.—For we must repeat, the shorter the time a given quantity of blood be drawn in, the greater will be the advantage resulting from it; hence I have, in several instances, bled in both arms at once." 321.

Temporal arteriotomy Dr. Dewes does not commend, as he thinks a sufficient quantity of blood cannot be quickly abstracted in this way.

"When blood has been freely drawn previously, and more is still wanting, then opening the temporal artery may be highly advantageous; but this operation, I think, should never be relied on, where much blood is necessary to be drawn." "Topical bleedings, by cupping and leeches, should never be had recourse to in the beginning of this disease, for the reasons we have urged against the gradual subtraction of blood."

In respect to the quantity of blood which it may be necessary to draw off, it must be determined by the effect produced.

"I bleed (says our author) until I abate the severity of the fits, or until I arrest their repetition. This may be effected sometimes by thirty or forty ounces suddenly drawn; but it may require upwards of an hundred in the course of a few hours. Beside bleeding generally and topically, other evacuations are to be promoted, such as purging, enemata of stimulating ingredients, sinapisms, blisters, &c." 322.

In respect to the hysterical form of puerperal convulsions, our author states that the cure is much more simple and certain than in the other species, and is to be conducted on precisely the same principles as though the woman were not pregnant at all. He has never considered delivery as essential to the welfare of the patient, unless it attacks where the process is pretty far advanced, and seems to arise from the irritation given by the head of the child suddenly to the os uteri; in this case to deliver may be important, as it immediately removes the cause of the convulsions. This form of the disease rarely requires more than one bleeding, and that not very large.

"After we have taken away blood, which should always be done when the pulse is full or tense, we may safely exhibit opium with assafœtida, which will generally pretty speedily arrest the disease. I have never found cupping or blistering necessary; having the bowels opened by injections is important. It was the treatment for this form of the disease which has given rise to almost all the errors in the management of puerperal convulsions. It was successfully treated by opium and antispasmodics; and as every other species was confounded with this, the same plan was pursued with them to the inevitable destruction of the patient." 331.

*Treatment of the Apoplectic Species.* There is no difference between the treatment of this species and the epileptic, except that blood-letting should, if possible, be more promptly employed, and more extensively used in the apoplectic form —"for if an hour be lost, the patient's doom may be sealed." —Even when most energetically treated, this difease is too often fatal.

"And we may add, as it forms a distinction between these two species, that there is one state of the patient in which artificial delivery is not to be thought of. But as this is a matter which should be clearly understood, we will state the treatment more at length. In order to do this with the least possible ambiguity, we shall divide this species into two varieties, and for want of better terms shall call one idiopathic, and the other symptomatic. By the former we wish to be understood that attack of convulsions, in which pregnancy or labour has no agency in the production of; and by the latter, that attack of convulsions which happens during the progress of labour, but in which this process had no other agency than producing a strong determination to the head. In variety first, we have seen a disease seize a pregnant woman without this state contributing especially to it; for if the same plethoric condition of the blood vessels should be produced without the circumstance of pregnancy, the same result would follow. In this variety then, we shall find that the premonitory symptoms preceded the attack but a short time—that they were more intense—but the convulsions are perhaps less severe, but more obstinate in their continuance, and less regular in their return; the breathing is more strongly stertorous, or is rather a loud snoring—that there is no change made in the os tincæ, nor any evidence of uterine contraction: in a word, not a symptom of labour. Here then, should we attempt delivery by forcing the mouth of the uterus, as some direct, we should inevitably destroy our patient; delivery in this case is not to be thought of, because there is no effort of nature for this object; and where this effort does not manifest itself, it were madness; nay I had like to have said, *murderous* to attempt it. Our whole duty, in this case, consists in proper medical treatment, and differs in no way from that we have already suggested for species the first, except in *this* case, the remedies require a more prompt and more extensive application. Effusion but too often takes place, and all our hopes are blasted in a moment. We may here observe, once for all, that the rules for the delivery of a patient labouring under convulsions, are simple, clear, and void of all ambiguity—they are these:

"1mo. When there is an evident disposition in the uterus to effect the expulsion of its contents, it is then, and then only, we are to attempt to assist it.

"2ndo. That this assistance must be given to the efforts of nature, with the least possible violence.

"3tio. That unless the labour be far advanced, and the delivery can be very promptly effected, either by turning or the forceps, it should not be attempted, until we have lessened the danger of a fatal effusion by a copious bleeding.

"4to. That no attempt should be made to dilate the mouth of the womb when at all rigid, until we have removed, or very much lessened the determination to the head by a *sufficient loss of blood.*

"5to. That this sufficient loss of blood is only manifested by a cessation, or a great abatement of the convulsions, or by an easy dilatability of the os tincæ.

"6to. That when the former condition obtains, we may safely trust to the efforts of nature to effect the latter; but if it be accompanied by the latter, the more speedily we deliver the patient the better.

"7mo. That turning is the means to be employed where the child is still enveloped in the uterus; but when the head has escaped from this viscus, we must employ the forceps." 327.

Dr. Dewes illustrates the principles laid down in this paper by a great number of cases; but these we do not deem it necessary to analyse, as we think the practical application of the above principles is sufficiently obvious, without occupying any farther space. The long obstetric experience of our author, in a land so celebrated for the flourishing state of its *puerperal* manufactories, entitles his observations to the respectful attention of his brethren in the "old world," where they will doubtless be favourably received. The following observations relative to the practice adopted in cases of puerperal convulsions here, are furnished us by an enlightened physician of this Metropolis, who has ample experience in a large Establishment for Parturient Females, as well as an extensive private practice.

"The treatment of the true, or epileptic form of puerperal convulsions is simple and effectual, because it is founded upon principles obvious, and intelligible. The symptoms and consequences of this formidable affection prove the existence of congestion in the vessels of the brain, attended commonly with general plethora. The remedies employed are consequently such as either directly or indirectly diminish vascular fulness, and relieve more especially the vessels of the head.

"Thus upon the appearance of this 'accidental occurrence,' it is the established practice at once to have recourse to sudden, large, and even repeated detractions of blood; proportioned, however, to the habit and state of health of the patient, to the severity and duration of the paroxysms, and to the continuance of that state of stupor, (extending as it often does to the heart and arteries, and reducing greatly the frequency of the pulse,) which generally succeeds the fits. Less than from 24 to 30 oz is seldom effectual, and more than from 70 to 100 oz. will be rarely required. It does not appear to be of much moment from what vessel the blood is drawn. It flows too slowly from the temporal artery to make that a desirable source, and the sudden and violent contortions of the body make the opening of the jugular vein a positively dangerous operation. Bleeding from the arm is the mode of venesection most easy of application, and it is equally effectual with any other.

"This then is the 'summum remedium,' without which recovery is almost miraculous, and from the due application of which we may with great confidence anticipate the patient's recovery. Practitioners, however, do not confine themselves exclusively to this single and singularly powerful remedy; it may be aided in its operation by other means, unsatisfactory indeed as principals, but valuable as auxiliaries in the cure of this disease. Such are the elevation of the head and shoulders above the level of the rest of the body; the removal of all compressing ligatures from the neck; local bleeding by leeches or cupping, where general bleeding has been carried as far as is justifiable; the application of cold spirituous, and, consequently, evaporating lotions, or what is better, ice enclosed in a bladder, and allowed to melt gradually upon the *shorn* head and along the course of the carotids. These are the subsidiary measures which by their DIRECT agency diminish the cerebral congestion—others INDIRECTLY tend to the same object by increasing the flow of blood in other directions, as blisters to the nape of the neck, purgatives by the mouth aided by stimulant clysters, and lastly, delivery, which seems to act only by removing the pressure from the external iliac arteries, and thus allowing an increased quantity of blood to be sent suddenly to the extremities; whereby the quantum of circulating mass remaining the same, less must necessarily pass by the carotids. It is upon this principle that fainting is often occurring after very rapid delivery, or the sudden removal by tapping of dropsical accumulations in the abdomen.

"The importance of delivery in puerperal convulsions appears to have been greatly over-rated; for it is now found by multiplied experience, that where it has been confided in to the exclusion of other remedies, the fits have generally returned during or after its accomplishment, and the patient has died:—and, e converso, where early and large bleeding, with those other means that have been noticed, have been adopted, the mother has commonly recovered, and the child not unfrequently been born alive, although the labour has been allowed to terminate without instrumental aid.

"Since then delivery is but a secondary, subsidiary, and indirect measure; as a general rule, we ought not to have recourse to it where it can endanger the child's life. This we are never justified in sacrificing, unless it contribute essentially to the preservation of the mother; and, in convulsions, it is at best but a questionable remedy. But there are cases in which we may, do, and perhaps ought to deliver, and it is an inquiry worth instituting, under what circumstances we should effect it; and upon this point our determination will be influenced by the presentation and stage of the labour.

"In head cases, previously to the dilatation of the os uteri, the labour is not to be interfered with, for artificial dilatation adds little or nothing to the chance of preserving the child; it is an operation painful, and not altogether devoid of danger to the mother; and above all, it has been found, that the paroxysms are reproduced or rendered more severe by every attempt to introduce the hand into the uterus.

"Where the orifice of the uterus is dilated, and the head of the child has *not* descended into the cavity of the pelvis, but remains loose and moveable at its upper aperture, it has been recommended to turn, and deliver footling; but this practice has been nearly, if not entirely, relinquished, in consequence of the attempt so frequently reproducing the fits. Besides, the cases are extremely rare in which, when the os uteri is completely or even nearly dilated, the head has not descended so low into the pelvis as to render the operation of turning impracticable, or at least very dangerous. Under such circumstances, where turning is impossible or unjustifiable, and yet the head has not descended so low as for the ear to be felt, the perforator has been used by some; the long forceps by a few; whilst the majority of practitioners defer the delivery till the head is come within reach of the short forceps. If the estimate we have formed of the value of delivery as a remedy for convulsions be just, we are clearly not authorised to use any instrument necessarily destructive of the child's life—the perforator therefore should not be employed; at all events not until the other remedies have been fairly tried and have failed. Those who are adroit in the application of the long forceps may employ them—but they are in the hands of few—are dangerous when applied by those unaccustomed to their use, and experience has shown that little, if any, inconvenience arises from waiting till the pains have pushed the head so low as that the ear may be felt. When the labour has advanced thus far, inasmuch as the delivery can be accomplished with perfect safety both to mother and child, we are justified in effecting it; rather, however, as a matter of choice, than of absolute necessity; we deliver because we can, rather than because we must,

Like purgatives and blisters, however, it may do good by contributing to the general result; and being usual in such cases, it is expected, and we would therefore urge its adoption. For this purpose the forceps are decidedly preferable to the lever, which is, in cases of convulsions, a very dangerous instrument. It is within the knowledge of the author of this summary of the treatment of convulsions, that in the hands of one who has for many years been in the habit of employing the lever, the head has been brought through the vulva at a moment, when the paroxysm prevented the patient from remaining quiet. The perinæum has been torn, and the woman has died at the end of three or four days of sphacelus. It is probable, that with the more manageable instrument, the forceps, no such accident would have occurred.

"In other presentations, the practice varies very little from that of similar cases, unaccompanied by convulsions. In breech cases, no assistance is to be given till the orifice is dilated, and the groins within reach; we may then accelerate the birth of the child.

"In footling cases, we are not to hurry the delivery till the soft parts are prepared for the passage of the child through them without the risk of dangerous compression of the navel-string.

"In the true cross-birth, or presentation of the upper extremities, or of any other part of the trunk than the nates, we MUST turn—this necessity arising, however, not from the convulsions, but from the position of the child; and all we have to attend to is, as in common cases of such presentations, to select the proper period; viz. when the os uteri is dilated, or nearly so, and the bag unruptured. But where there are attending convulsions, we must be doubly cautious about artificial dilatation, and the introduction of the hand into a strongly contracting uterus, either of which is peculiarly apt to reproduce the fits.

"Thus, to recapitulate the leading points of treatment, it appears that the remedies in which British practitioners have the greatest confidence, in the cure of puerperal convulsions, are, bleeding, general and local; cold applied to the elevated and shorn head; blisters to the nape of the neck; purgatives; stimulant clysters; and lastly, delivery, where this can be accomplished without injury to the mother or danger to the child: and we shall only add, that since these means have been more sedulously and actively employed, the mortality from the disease has been in a corresponding ratio diminished, until at length the cases of fatal termination are bearing but a very small proportion to those of speedy and permanent recovery."

